# Physiology and Gene Expression Analysis of Potato (*Solanum tuberosum* L.) in Salt Stress

**DOI:** 10.3390/plants11121565

**Published:** 2022-06-14

**Authors:** Qing Li, Yuzhi Qin, Xinxi Hu, Liping Jin, Guangcun Li, Zhenping Gong, Xingyao Xiong, Wanxing Wang

**Affiliations:** 1 Institute of Vegetables and Flowers, Chinese Academy of Agricultural Sciences, Key Laboratory of Biology and Genetic Improvement of Root and Tuber Crops, Ministry of Agriculture, Beijing 100081, China; liqinghq@126.com (Q.L.); jinliping@caas.cn (L.J.); liguangcun@caas.cn (G.L.); 2 College of Horticulture, Hunan Agricultural University, Engineering Research Center for Horticultural Crop Germplasm Creation and New Variety Breeding, Ministry of Education Changsha, Hunan Provincial Engineering Research Center for Potatoes, Southern Regional Collaborative Innovation Center for Grain and Oil Crops in China, Key Laboratory for Vegetable Biology of Hunan Province, Changsha 410128, China; qyuz@163.com (Y.Q.); huxinxi@163.com (X.H.); 3 Tangshan Academy of Agricultural Sciences, Tangshan 063001, China; gong093@163.com; 4 Agricultural Genomics Institute at Shenzhen, Chinese Academy of Agricultural Sciences, Shenzhen 518120, China

**Keywords:** physiology, phenotyping, ion flux, ion transport genes, transcription factors

## Abstract

The production of potato (*Solanum tuberosum* L.) faces a severe challenge due to the salinization of arable land worldwide. The cultivation of salt-tolerant potatoes is of great significance to ensure food security. In this study, two cultivars of ‘Longshu 5’ and ‘Qingshu 9’ were compared for physiological responses to salt stress, and then the salt tolerance of the two cultivars were assessed via principal component analysis. Furthermore, the Na^+^, K^+^, and Ca^2+^ flux of the cultivars under salt stress was recorded. Finally, the expression levels of ion transport-related genes and transcription factors in salt-tolerant cultivars were explored under NaCl stress. The results showed that the seven physiological indicators of salt tolerance were differed between the cultivars. Interestingly, soluble protein and sugar were early responsive to salt stress than proline in the salt-tolerance cultivar. Peroxidase and superoxide dismutase activity were significantly different in ‘Longshu 5’ under NaCl stress and without being significantly different in ‘Qingshu9’. In addition, the salt tolerance of ‘Longshu 5’ was more tolerant than ‘Qingshu 9’ based on principal component evaluation. Meanwhile, the strong efflux of Na^+^, the stability of K^+^, and the high absorption of Ca^2+^ in ‘Longshu 5’ indicated salt adaption mechanisms in the salt-tolerant potato. In addition, we found that ion transport-related genes and transcription factors, such as *StSOS1*, *StNHX4*, *StAKT1*, *StNAC24*, and *StCYP707A*, played a role in the salt tolerance of ‘Longshu 5’. In conclusion, the salt-tolerant potato can regulate physiological substances to adapt to salt stress, and ion transport related genes and transcription factors play a role in improving salt tolerance.

## 1. Introduction

Salt is an important abiotic factor affecting plant growth and secondary metabolism [1]. Faried et al. [2]. reported that soil salinization negatively affected the growth and yield of potato crops, especially under arid and semiarid climate. Soil salinization has developed into a global concern. With increasing salinization, grain yield will be affected, and food security will face severe challenges. Potato is the fourth major crop in the world; however, salt stress can lead to serious declines in potato yield [3]. Salinity causes stress effects for the whole plant with the induced secondary injury [4]. The main reason that plants suffer from salt damage is that a large amount of Na^+^ enters plant cells [5] and decreases plant capacity to extract water from soil, which determines the accumulation of ions like Na^+^ and Cl^−^ at toxic concentrations in cell tissues. This dual osmotic and ionic stress reduces cell and tissue expansion and causes nutritional imbalance, osmotic stress, and oxidative stress, affecting plant growth, development, and survival [6]. Fortunately, plants have evolved defense mechanisms, such as producing compatible solutes, antioxidant stress, and resisting membrane lipid oxidation, to adapt to salt stress [7].

Plants take specific measures, such as accumulating the high concentrations of osmosis-regulating substances in leaves, to minimize salt-stress damage [7]. A previous study showed that tissue culture seedlings of potato can accumulate proline under different salt gradients [8]. In the stem tissue of potato, the tolerant cultivars accumulated less H_2_O_2_ and more proline when compared to the susceptible varieties [9]. However, salinity stress caused a significant accumulation of H_2_O_2_ and proline in salinity-sensitive barley [10]. These studies indicate that the same osmoregulatory substances may differently respond to salt stress in different species. Salinity stress decreased the starch content but increased the sucrose content in *Coreopsis tinctoria* Nutt [11]. These compatible solutes play an important role in adapting to salt stress. In addition, with the plant-exposed salt environment, reactive oxygen species (ROS) with high activity and toxicity are produced [8]. ROS cause membranous peroxidation [12], inducing increased malondialdehyde (MDA) content that is a hallmark of membranous peroxidation [13]. In turn, the expression of antioxidant defense genes become triggered to increased activities of antioxidant enzymes, such as superoxide dismutase (SOD), peroxidase (POD), and catalase (CAT), to defend the cell against salt stress [14].

Potato has been classified as a moderately salt-sensitive crop. However, variations in salt sensitivity among various cultivars of potato have been observed [15,16]. The salt tolerance of a few potato cultivars has been evaluated under field and greenhouse conditions [16]. Studies have shown that the salt-tolerant materials identified in vitro were also salt-tolerant in field planting. The results showed that the two identification results were correlated [17]. Due to the long growth cycle and complex environment of potatoes in the field, the field identification is time-consuming and labor-intensive when a large number of materials need to be identified for the salt tolerance. In vitro evaluation of salt stress is an alternative to costly, labor-intensive, and occasionally problematic field-based evaluations [18]. Although the indoor evaluation period is short, it usually takes about 4 weeks, with low efficiency [19]. In addition, growth parameters, such as production, are commonly used to evaluate the salt tolerance of plants [20]. Physiological and biochemical indicators, such as proline, protein, sugar, SOD, POD, and CAT, are rarely used to assess salt tolerance in potato. Furthermore, complex and important traits, such as salt tolerance, might be difficult to dissect, and a simple pairwise analysis may not fully reflect the true salt tolerance of the plants [8]. In this case, data can be analyzed using principal component analysis (PCA) [19]. PCA is a statistical method of simplifying a dataset. In order to comprehensively and systematically analyze problems, we must consider many influencing factors (indicators or variables). Since each index reflects some information of the research problem to a different extent, each have a certain correlation between indicators. Thus, they reflect a certain degree overlapping information. Principal component analysis not only preserves the main information of the original variables, but also simplifies the data [21]. Therefore, PCA can be used to simplify biochemical indexes and identify complex salt-tolerance traits in potato.

The resistance or adaptation of plants to external salt stress tends to be Na^+^ efflux and the localization of vacuoles [22]. In addition, the increase of osmotic regulation substances and antioxidant substances, the high expression of ion transport-related genes, and transcription factors are effective ways to enhance salt tolerance [8]. Salt overly sensitive (SOS) is a Ca^2+^ signal-dependent ion transport pathway that regulates the salt-stress response in plants [5] and is one of the most important salt-tolerance pathways. SOS1 (a membranous Na^+^/H^+^ reverse transporter) is responsible for excreting excess Na^+^ from cells. *AtNHX* (a possible vacuolar Na^+^/H^+^ exchanger) is responsible for the localization of Na^+^ within vacuoles in Arabidopsis thaliana [23]. In order to counter the toxic effects caused by Na^+^, the input of K^+^ is very important. The potassium channel KAT1 and the AKT1 signaling module in Arabidopsis synergism promotes K^+^ influx, leading to a decrease in the Na^+^: K^+^ ratio [24]. The NAC transcription factor family have been shown to be involved in abiotic stress response [25]. Comparative RNA sequencing (RNA-seq)-based analysis revealed that the expression of 43 NAC genes was up- or downregulated under salt stress and under drought stress [26]. Because both drought stress and salt stress lead to osmotic stress to some extent, plants may have common resistance mechanisms to the osmotic stress [27]. The results showed that drought and salinity tolerance have common tolerance mechanisms. Transcription factor VIP1 is important in hyperosmosiation-induced ABA degradation and salt-stress-induced MAPK signaling pathway [28]. Moreover, 14-3-3 is involved in VIP1 phosphorylation and dephosphorylation as well as the ion transport pathway [29]. Therefore, it is speculated that transcription factor VIP1 may play a role as a bridge between the MAPK signaling pathway and the ion transport pathway.

At present, the commercial potato cultivars show promising differences in salt tolerance [30]. In a previous study, it was found that the phenotype (the individual trait was exhibited) of ‘Longshu 5’ under salt stimulation (500 mmol/L) did not change significantly compared with that of the control group. It indicated that ‘Longshu 5′ was tolerant to salt stress. However, under long-term salt stress or short-term salt shock, the growth of ‘Qingshu 9’ (it was reported that the cultivar was tolerant to drought) [31] was inhibited, with severe wilting [19]. To study the physiological response of the two cultivars to salt shock, the changes of compatible solutes, antioxidants, and lipid peroxidation were compared in the present study. The salt tolerance of different materials was evaluated under continuous, high-concentration salt (NaCl) shock. The ion flux trends in the meristem zone of potato were analyzed. Finally, the role of the ion transport-related genes in the salt-tolerance cultivar under NaCl treatment were explored. The results of this study will offer valuable information for evaluating the salt tolerance of potato. Meanwhile, understanding the physiological and molecular mechanism of the salt-tolerance potato will provide cultivar resources and theoretical basis for breeding and screening salt-tolerant potato varieties.

## 2. Results

### 2.1. Screening the Appropriate Salt Concentration for Physiological Measurements

Salt concentration screening experiment showed that the two cultivars can take root at 0–100 mmol/L with 100% rooting percentage, while no rooting was observed at other concentrations. The fresh weight decreased with the increase of the salt concentration, and the total coefficient of variation (CV) of the root percentage and the fresh weight reached the maximum of 122.1% at 500 mmol/L. Moreover, all plants were wilted at 700–900 mmol/L, showing that the concentration range was not suitable for the growth of potato tissue culture seedlings. The wilt rate of ‘Qingshu 9’ (33.33%) was higher than that of ‘Longshu 5’ (26.67%) at 500 mmol/L, and they were not different at 0–300 mmol/L, indicating that 500 mmol/L NaCl was an appropriate salt shock concentration for physiological measurements within 96 h (Table 1).

### 2.2. Proline, Soluble Protein, and Soluble Sugar Respond Differently to Salt Stress

**Figure 1:**
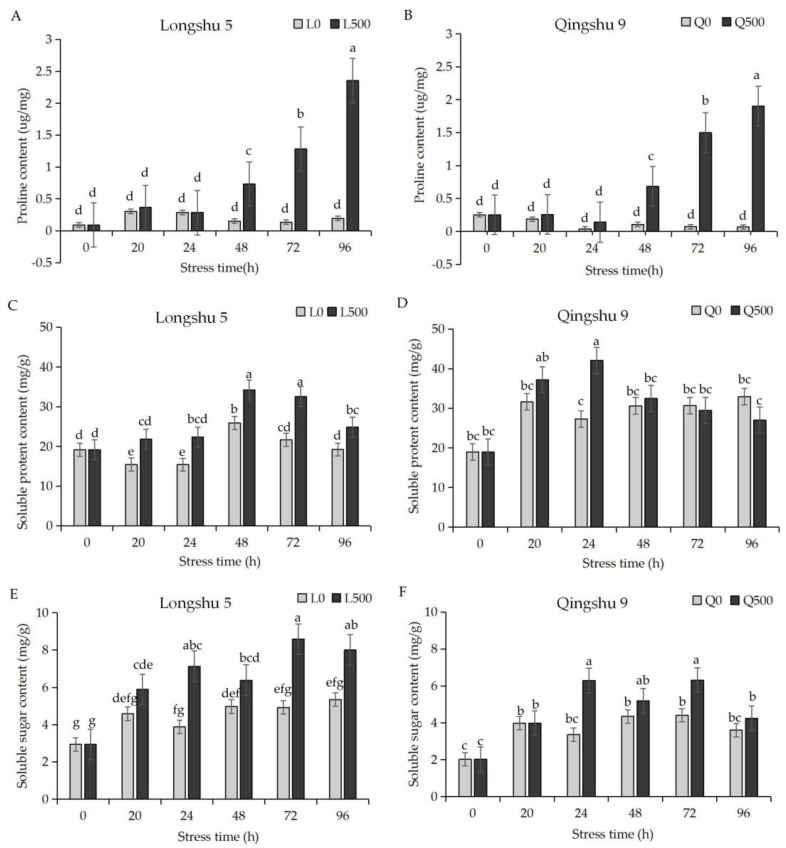
Proline, soluble protein, and soluble sugar responses to salt stress. “L0” and “L500” represented Longshu5 and was exposed to 0 mmol/L and 500 mmol/L NaCl stress, respectively. “Q0” and “Q500” represented Qingshu9, with exposure to 0 mmol/L and 500 mmol/L NaCl stress, respectively. (**A**) ‘Longshu 5’ proline content, (**B**) ‘Qingshu 9’ proline content, (**C**) ‘Longshu 5’ soluble protein content, (**D**) ‘Qingshu 9’ soluble protein content, (**E**) ‘Longshu 5’ soluble sugar content, and (**F**) ‘Qingshu 9’ soluble sugar content were measured at 0, 20, 24, 48, 72, and 96 h. Gray and black columns show 0 and 500 mmol/L NaCl concentrations, respectively. Values are means and bars indicate SDs (*n* = 3). Same lowercase letters mean no significant difference, different lowercase letters mean significant difference.

Under 500 mmol/L NaCl, the proline content of ‘Longshu 5’ and ‘Qingshu 9’ increased compared with the control. The increase was small at 20–24 h, but increased sharply at 48, 72, and 96 h; for ‘Longshu 5’, the increases were 4.8-, 9.5-, and 13.1-fold, respectively (Figure 1A), and for ‘Qingshu 9’, the increases were 6.5-, 20.4-, and 26.5-fold, respectively (Figure 1B). The total content of proline at 96 h was higher in ‘Longshu 5’ than ‘Qingshu 9’.

The soluble protein content first increased and then decreased. The increase of soluble protein content in ‘Longshu 5’ was 50.10% at 72 h, and 29.50% at 96 h (Figure 1C). Soluble protein content in ‘Qingshu 9’ increased by 81.70% at 24 h and decreased by 4.1% and 24.80% at 72 h and 96 h, respectively. With a longer stress time, compared with the control, the soluble protein content of ‘Qingshu 9’ was decreased (Figure 1D).

Under salt stress, the soluble sugar content of ‘Longshu 5’ and ‘Qingshu 9’ increased compared to the control, with similar trends. Their increases reached the maximum at 24 h, with 96.70% for ‘Longshu 5’ and 100.8% for ‘Qingshu 9’. At other time points, the increase of ‘Longshu 5’ was higher than that of ‘Qingshu 9’. The soluble sugar content of ‘Longshu 5’ was significantly higher than that of the control at 96 h, while ‘Qingshu 9’ was not significantly different from the control (Figure 1E,F).

### 2.3. Antioxidant Enzyme Activities and MDA Respond Differently to Salt Stress

**Figure 2:**
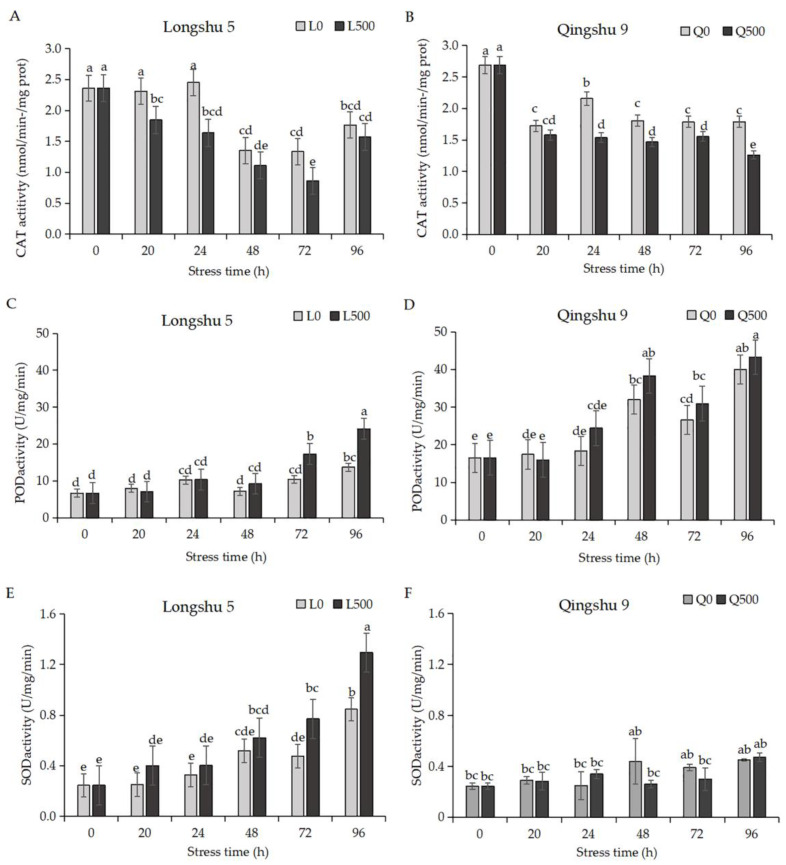
Antioxidant enzyme activity responses to salt stress. “L0” and “L500” represent Longshu 5 exposed to 0 mmol/L and 500 mmol/L NaCl stress, respectively. “Q0” and “Q500” represent Qingshu9 exposure to 0 mmol/L and 500 mmol/L NaCl stress, respectively. (**A**): ‘Longshu 5’ CAT activity, (**B**): ‘Qingshu 9’ CAT activity, (**C**): ‘Longshu 5’ POD activity, (**D**): ‘Qingshu 9’ POD activity, (**E**): ‘Longshu 5’ SOD activity, and (**F**): ‘Qingshu 9’ SOD activity were measured at 0, 20, 24, 48, 72, and 96 h. Gray and black columns show 0 and 500 mmol/L NaCl concentration, respectively. Values are means and bars indicate SDs (*n* = 3). Same lowercase letters mean no significant difference, different lowercase letters mean significant difference. CAT, catalase; POD, peroxidase; SOD, superoxide dismutase.

Salt stress induces oxidative stress in plants and the antioxidant capacity of plants can be expressed by the activity of antioxidant enzymes. Under salt stress, the CAT activity of ‘Longshu 5’ significantly decreased by 33.24% and 35.45% at 24 h and 72 h, respectively, and was still lower than that of the control at 48 h and 96 h, but the differences were not significant (Figure 2A). CAT activity in ‘Qingshu 9’ decreased significantly by 28.88%, 18.82%, 12.87%, and 29.51% at 24, 48, 72, and 96 h, respectively (Figure 2B). Compared to the control, the POD activity of ‘Longshu 5’ increased by 28.80%, 66.14%, and 76.05% at 48, 72, and 96 h, respectively, and the differences were significant at 72 h and 96 h (Figure 2C). The increase of POD activity of ‘Qingshu 9’ was not significant (Figure 2D). The SOD activities of ‘Longshu 5’ and ‘Qingshu 9’ were significantly different from that of the control under salt stress. The SOD activity of ‘Longshu 5’ was higher than that of the control at each time point and increased to a significant level at 72 and 96 h, with increases of 62.32% and 52.89%, respectively. Compared with control, SOD activity of ‘Qingshu 9’ was not significantly different (Figure 2E,F). Therefore, the increase of POD and SOD activities of ‘Longshu 5’ were significantly higher than those of ‘Qingshu 9’ under NaCl stress.

MDA is the product of membrane peroxidation, and its content can indirectly indicate the extent of membrane peroxidation [32]. Under salt stress, the content of MDA in ‘Longshu 5’ increased slowly but the difference from the control was significant at 72 h and 96 h, with maximum increases of 1.81 and 3.78 nmol/g, respectively. The MDA content of ‘Qingshu 9’ increased first and then decreased, but it increased significantly at 48 h compared with the control (0 mmol/L NaCl), with an increase of 3.92 nmol/g (Table 2). Therefore, the membrane damage of ‘Qingshu 9’ was more severe and occurred earlier than that of ‘Longshu 5’.

### 2.4. Principal Component Analysis of Salt Tolerance of Different Cultivars

Seven indicators were transformed to the salt-tolerance coefficient (Table S1) and then standardized via Zscore. The correlation matrix showed that there was information overlap among these indicators: CAT activity was differently negatively correlated with proline content and soluble sugar; SOD activity had a highly significant positive correlation with soluble protein (Table S2).

In addition, the principal component analysis was implemented for comprehensive assessment of the salt tolerance of cultivars. According to initial eigenvalues greater than 1, the first three principal components were extracted. Component 1 explained 44.912% of the variation, and the three components together explained 84.062% (Table 3). The characteristic vector transformed from the component matrix represented the correlation coefficient between principal components and corresponding variables (Table S3 and Table 3).

Therefore, the three new variables were linear combinations of the original seven variables, and the three principal component formulas can be expressed as below:

PCA 1 = 0.095X1 + 0.395X2 + 0.483X3 − 0.345X4 + 0.421X5 + 0.41X6 + 0.369X7(1)

PCA 2 = −0.728X1 + 0.479X2 − 0.006X3 + 0.315X4 − 0.245X5 + 0.281X6 − 0.055X7(2)

PCA 3 = −0.049X1 − 0.263X2 − 0.131X3 + 0.629X4 + 0.337X5 + 0.03X6 + 0.633X7(3)

**Figure 3:**
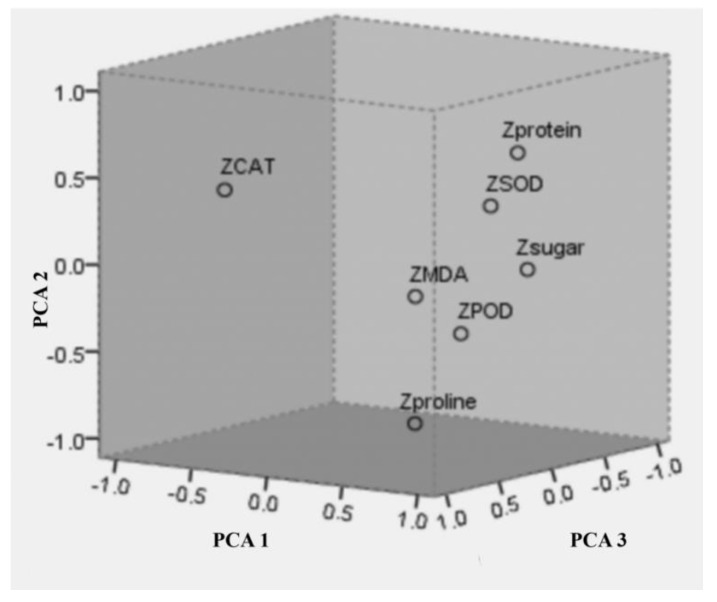
Three-dimensional load diagram of seven indicators. Z represents that the analysis is performed using normalized data. CAT, catalase; POD, peroxidase; SOD, superoxide dismutase; MDA, malondialdehyde; PCA, principal component.

The three-dimensional load plot showed that the soluble sugar contributed most to component 1, while proline and MDA mainly contributed to component 2 and component 3, respectively (Figure 3). Finally, the composite scores were calculated via the comprehensive evaluation function: composite scores = 0.449 PCA 1 + 0.245 PCA 2 + 0.146 PCA 3 (0.449, 0.245, and 0.146 were percentage of variance in Table 3). PCs and composite scores indicated that the salt tolerance of ‘Longshu 5’ was stronger than ‘Qingshu 9’, with a high total score (1.703) (Table 4).

### 2.5. Na^+^, K^+^, and Ca^2+^ Flux of Potato Root under Salt Stress

**Figure 4:**
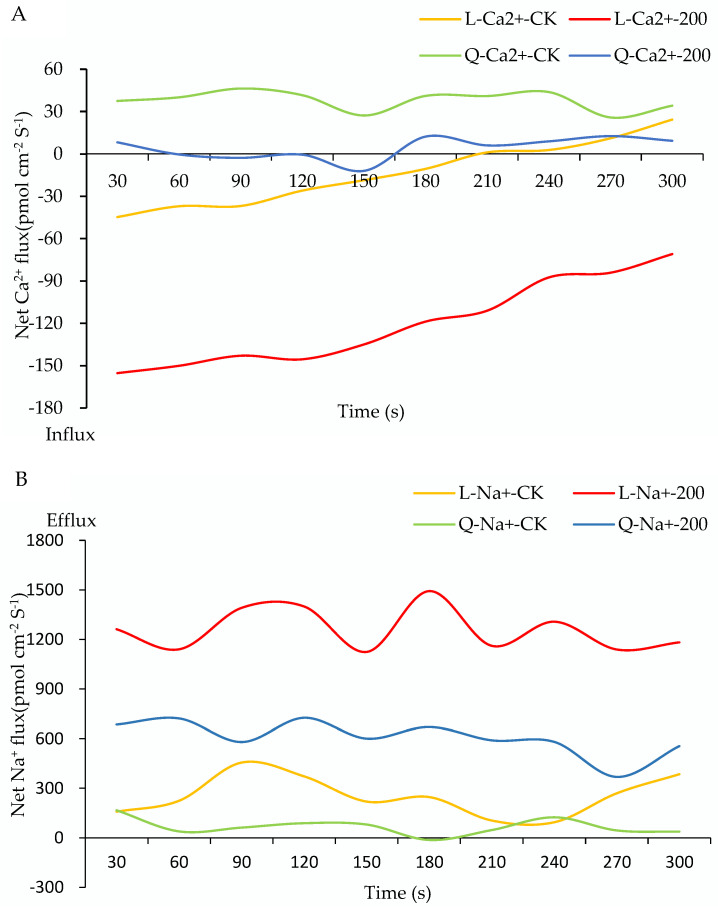

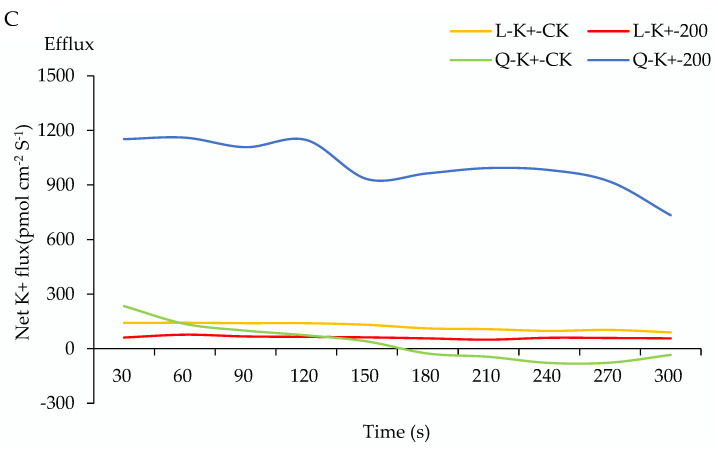
Ion flux analysis of root meristem of two potato cultivars. The net ion flux (the flux offset by the efflux and influx) was obtained. (**A**–**C**) represented the net flux of Ca^2+^, Na^+^, and K^+^, respectively. L-Ca^2+^-CK: The net Ca^2+^ ion flux was observed in ‘Longshu 5’, which was grown in Murashige and Skoog (MS) medium without NaCl stress; L-Ca^2+^-200: The net Ca^2+^ ion flux was observed in ‘Longshu 5′, which was grown in MS medium with 200 mmol/L NaCl stress; Q-Ca^2+^-CK: The net Ca^2+^ ion flux was observed in ‘Qingshu 9′, which was grown in MS medium without NaCl stress; Q-Ca^2+^-200: The net Ca^2+^ ion flux was observed in ‘Qingshu 9’, which was grown in MS medium with 200 mmol/L NaCl stress; L-Na^+^-CK: The net Na^+^ ion flux was observed in ‘Longshu 5’, which was grown in MS medium without NaCl stress; L-Na^+^-200: The net Na^+^ ion flux was observed in ‘Longshu 5′, which was grown in MS medium with 200 mmol/L NaCl stress; Q-Na^+^-CK: The net Na^+^ ion flux was observed in ‘Qingshu 9’, which was grown in MS medium without NaCl stress; Q-Na^+^-200: The net Na^+^ ion flux was observed in ‘Qingshu 9’, which was grown in MS medium with 200 mmol/L NaCl stress; L-K^+^-CK: The net K^+^ ion flux was observed in ‘Longshu 5’, which was grown in MS medium without NaCl stress; L-K^+^-200: The net K^+^ ion flux was observed in ‘Longshu 5’, which was grown in MS medium with 200 mmol/L NaCl stress; Q-K^+^-CK: The net K^+^ ion flux was observed in ‘Qingshu 9’, which was grown in MS medium without NaCl stress; Q-K^+^-200: The net K^+^ ion flux was observed in ‘Qingshu 9’, which was grown in MS medium with 200 mmol/L NaCl stress. Data shows ion flow within 300 s. The positive value represented a net ionic efflux; The positive value represented a net ionic influx.

In order to explore the mechanism of strong salt tolerance in ‘Longshu 5’, the Ca^2+^, Na^+^, and K^+^ flux of ‘Longshu 5’ and ‘Qingshu 9’ were measured in the root meristem zone with or without NaCl stress for 6 h. The Ca^2+^ net flux (fluctuation from −200 to −100) in ‘Longshu 5’ under NaCl stress was less than that of the control (fluctuation from −50 to 50), whereas there were small differences in ‘Qingshu 9’, with a fluctuation from 0 to 25 under salt stress and from 25 to 50 without salt stress (Figure 4A), indicating a stronger Ca^2+^ uptake of ‘Longshu 5’ than in ‘Qingshu 9’. The Na^+^ net fluxes of both cultivars were higher under NaCl than without NaCl, with the fluctuation value of 1100 in ‘Longshu 5’ and fluctuation value of 700 in ‘Qingshu 9’ (Figure 4B), which indicated that the Na^+^ excretion of ‘Longshu 5’ was stronger than ‘Qingshu 9’. The K^+^ net fluxes were equivalent in ‘Longshu 5’ with or without NaCl stress, with the fluctuation value of 100, whereas the K^+^ net flux of ‘Qingshu 9’ (fluctuation from 800 to 1300) under NaCl stress was significantly higher than that of the control (fluctuations from −100 to 300) (Figure 4C), which showed that the stability of K^+^ can be maintained in ‘Longshu 5’, while there was K^+^ efflux in ‘Qingshu 9’ under salt stress.

### 2.6. Expression Analysis of Salt-Response-Related Genes and Transcription Factors under NaCl Treatment in a Time Course

**Figure 5:**
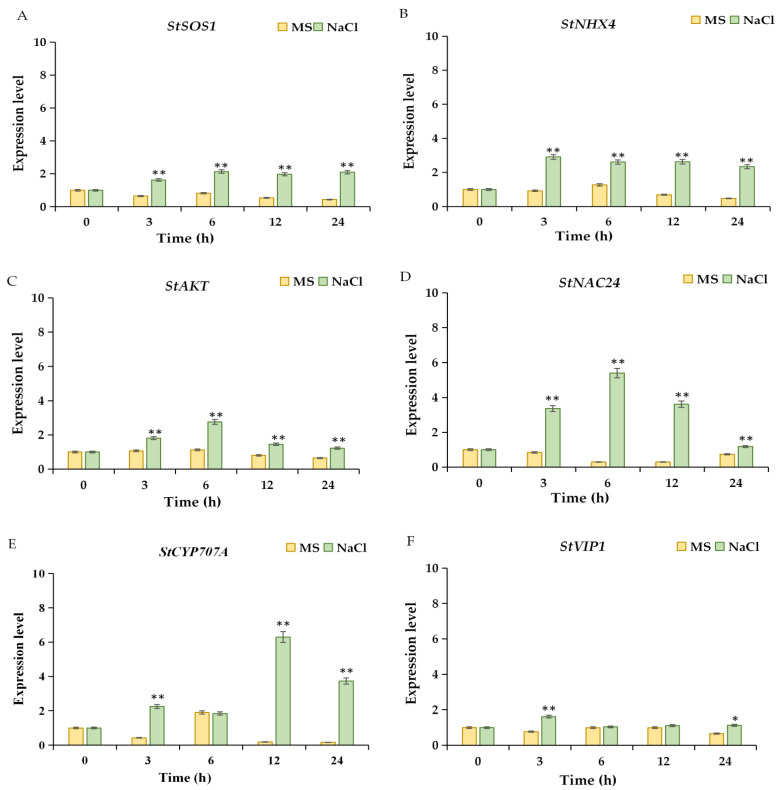
The expression level of ion transport-related genes and transcription factors under NaCl stress within 24 h. MS indicated that potatoes were grown in Murashige and Skoog medium without NaCl stress. NaCl indicated that potatoes were grown in Murashige and Skoog medium with 200 mmol/L NaCl stress. (**A**–**F**) were the expression levels of *StSOS1*, *StNHX4*, *StAKT*, *StNAC24*, *StCYP707A*, and *StVIP1*, respectively. * and ** represent significant and very significant differences, respectively. StSOS1, a membranous Na^+^/H^+^ reverse transporter 1; StNHX4, a possible vacuolar Na^+^/H^+^ exchanger 4; StAKT, K^+^ transporter; StNAC24, NAC transcription factor 24; StCYP707A, (+)-Abscisic Acid 8′-Hydroxylase; StVIP1, Vire2-interacting protein 1.

In order to investigate the expression levels of ion transport-related genes and transcription factors in the salt-tolerant cultivar ‘Longshu 5’ in MS and NaCl treatment in 0 h, 3 h, 6 h, 12 h, and 24 h, and different concentrations of NaCl in 24 h, total RNA were extracted, and qRT-PCR were implemented. The qRT-PCR results showed that the expression levels of *StSOS1*, *StNHX4*, *StAKT1*, *StNAC24*, and *StCYP707A* were significantly increased within 24 h after the NaCl treatment (Figure 5A–C). The expression level of *StNAC24* after 6 h of NaCl treatment was 18.72 times that of the untreated group, and the expression level of *StCYP707A* after 12 h of NaCl treatment was 35 times that of the untreated group (Figure 5D,E). The expression level of *StVIP1* was significantly increased only in the NaCl treatment for 3 h and 24 h compared with the untreated group (Figure 5F).

**Figure 6:**
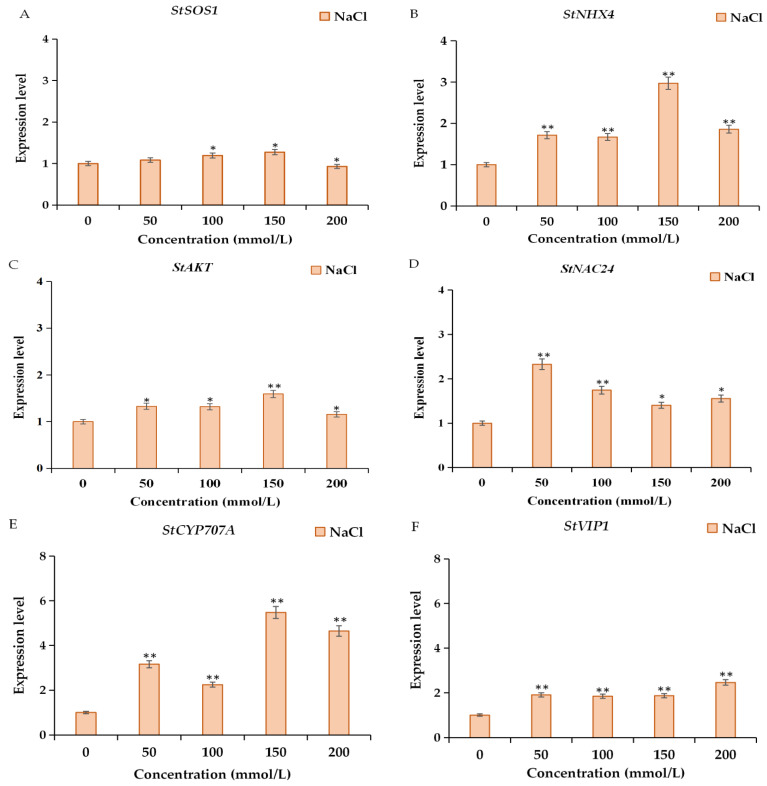
The expression level of ion transport-related genes and transcription factors under different concentration NaCl at 24 h. (**A**–**F**) were the expression levels of *StSOS1*, *StNHX4*, *StAKT*, *StNAC24*, *StCYP707A*, and *StVIP1*, respectively. * and ** represent significant and very significant differences, respectively. StSOS1, a membranous Na^+^/H^+^ reverse transporter 1; StNHX4, a possible vacuolar Na^+^/H^+^ exchanger 4; StAKT, K^+^ transporter; StNAC24, NAC transcription factor 24; StCYP707A, (+)-Abscisic Acid 8′-Hydroxylase; StVIP1, Vire2-interacting protein 1.

In addition, compared with the 0 mmol/L NaCl treatment, the expression levels of *StSOS1*, *StNHX4*, *StAKT1*, *StNAC24*, *StCYP707A*, and *StVIP1* were significantly increased under NaCl (Figure 6), especially the relative expression levels of *StNHX4* and *StCYP707A*, which were 2.97 and 5.48 times at 150 mmol/L (Figure 6B,E), respectively. These results suggest that ion transport-related genes and transcription factors play an important role in the salt tolerance of ‘Longshu 5’.

## 3. Discussion

Plants manage salinity stress injuries through a plethora of molecular, biochemical, and physiological changes, resulting in a remodulation of metabolic pathways to reach a new homeostatic equilibrium. These metabolites, nontoxic at high concentrations, are rapidly synthesized under salinity and rapidly removed (degraded or reallocated) when no longer required [33]. Proline, soluble proteins, and soluble sugars are important compatible solutes, and proline also functions as an antioxidant [34]. A report found that salt stress increased the content of proline in salt-tolerant and salt-sensitive potato [2]. The results in this study found that proline increased in the two cultivars, indicated that proline responded to salt stress, and thus proline can not accurately reflect the salt tolerance of varieties used in salt tolerance evaluation alone. Soluble protein in leaves of strawberry under salt stress is decrease or are not changed [35]. As saltiness increases the resistance of natural responses in this plant, it gets better at considering strategies for the increased production of proline [36] and sugar solution [37]. However, the soluble sugar content of potato under NaCl stress was observed to be increased at the early stages and decreased at late stages [38]. In ‘Qingshu 9’ (a drought tolerant variety), it was speculated that the main effect in the early stage of NaCl stress was osmotic stress, and ‘Qingshu 9’ increased soluble sugar to resist osmotic stress. The main effect in the later stage was ion stress. ‘Qingshu 9’ was sensitive to salt stress, resulting in the decrease of soluble sugar. Ye et al. [39] reported that the comparative proteomic analyses suggested that abundance of 33 proteins commonly increased in drought and salt stresses. Especially, carbon fixation pathways were commonly regulated. A study indicated that the compatible solutes, such as proline and carbohydrates, were effected by Na^+^ concentrations in the plant [40]. This study showed that salt stress induced the increase of soluble protein and soluble sugar in ‘Longshu 5’, but, at 96 h, the soluble protein of ‘Qingshu 9’ decreased. The change of soluble protein and sugar in ‘Qingshu 9’ illuminated that short salt shock caused the instantaneous increase of regulatory substances in the salt-susceptive cultivar, but limited the ability to regulate salt stress in the salt-susceptible cultivars. In addition, this study also found that the response of soluble protein and soluble sugar to salt stress occurred earlier than that of proline in potato. In this case, if in the productive system the cultivar is more susceptible to initial stress, select for soluble proteins, and if the material is more susceptible to late stress, select for proline.

Salt stress induces the accumulation of ROS, which have oxidative stress-induced toxic effects on plants. Besides their toxic effects, ROS also function as signaling molecules in response to environmental stimuli. ROS must be present at suitable levels in plant cells [41]. However, the boundary of ROS as toxic molecules and signaling molecules is not very clear. It is clear that plant cells can activate ROS as the second messenger only when their intracellular ROS levels are increased by external stimuli, such as salt stress and drought stress [5]. To combat salinity, plants have developed the adaptive defense mechanism of the ROS-scavenging system. Salinity stress caused a greater enhancement in antioxidant enzymatic activities at one specific time or in the tissues in tolerant barley [10], and salinity treatments significantly induced antioxidant enzyme activities, such as catalase, ascorbate peroxidase, guaiacol peroxidase, and superoxide dismutase, and enhanced antioxidant defense in salt-tolerant wheat [40]. A study has proved that increased antioxidant enzyme activity can enhance the salt tolerance of potato [30]. These studies found that SOD and POD activities were increased in salt-tolerance potato [42,43]. Our results are consistent with those of the predecessor. We found that the SOD and POD activities of Longshu 5 increased significantly, while the POD activity of Qingshu 9 did not increase significantly compared to the control. Interestingly, CAT activity was not increased in our study. However, the previous studies have shown that CAT activity was significantly increased in salt-tolerant potatoes [44]. We speculated that SOD and POD might play a major role in our salt-tolerant varieties. MDA is a product of membranous lipid peroxidation [45]. Many studies indicate that MDA increased in both the tolerant and sensitive lines with the increase of antioxidase under salt stress [10]. In the present study, the MDA content increased in the two cultivars. However, compared with the tolerant cultivar, the sensitive one accumulated greater MDA. Gao et al. [7] found that the activities of SOD and CAT may not be enough to eliminate ROS and resulted in the production of MDA under higher salt stress. Therefore, antioxidant enzymes can effectively reduce membrane lipid peroxidation, but they cannot prevent the production of MDA. MDA may play a signal molecular role to activate other salt-tolerance pathways. MDA was positively correlated with plasma membrane permeability, and a certain amount of MDA promoted the increase of plasma membrane permeability [8]. This means that the ions associated with salt stress, such as Ca^2+^, more easily penetrate the plasma membrane. Ca^2+^ signaling, as a second messenger, activates the salt sensitive (SOS) signal transduction pathway in salt-tolerant species [46]. In this case, the combined action between the tolerance mechanisms is indicated, as it occurs in most quantitative traits that have many genes involved and a high environmental effect. Based on the results of our study, we believe that under the same salt-stress conditions, varieties with lower MDA content are more tolerant to salt.

The growing problem of soil salinization has made the study of the salt-tolerance physiology of plants increasingly important. Researchers have explored the physiological response mechanisms of plants to salt stress [41]. However, due to the diversity and complexity of plant salt-tolerance pathways, a systematic salt-tolerance mechanism has not been proposed. In this case, productivity under stress conditions should be the main selection criterion, but a screening under the conditions of the present manuscript could be useful in a preselection process, to later be validated under field conditions. The response of physiological and biochemical indicators in a short time, and then using these responses to verify the salt tolerance of potato, are rarely reported. The most direct way to identify salt-tolerant cultivars is to conduct field tests on salt-bearing soils, but this requires a long time, a heavy workload, and a high investment, and it cannot meet the requirements of rapid and efficient mass evaluation [19]. Evidence indicates that there is a genetic basis for salt tolerance in cultivated potato [30]. In vitro culture is currently considered to be a low-cost alternative to the labor-intensive, time-, and space-limited assessment of salt tolerance in the field [19], and physiological indicators have significant differences between cultivars that can be used to identify plant salt tolerance [15]. Therefore, in this study, the in vitro culture method was used to identify the salt tolerance of potatoes through PCA. The results showed that the three components explained 84.062% of the variation and can represent the initial seven variables, and soluble sugar and proline contents had high contribution rates to PCs. Furthermore, the salt tolerance of ‘Longshu 5’, with a high total score, was superior to that of ‘Qingshu 9’, which is consistent with the result of the growth parameters identification in our laboratory. Under NaCl stress, Longshu5 grew better than Qingshu9 (Figure S1) [17]. Moreover, in the study, the evaluation measure was efficient, with just 96 h spent.

When plants are exposed to soil with high salinity causing ion toxicity due to the accumulation of toxic ions such as Na^+^ [47], reducing ion toxicity is an important strategy for plants to tolerate the salt stress. Plants perceive external salt-stress signals by salt sensors and develop ion tolerance by limiting the uptake of Na^+^ and reducing their transport. Finally, toxic ions are localized in vacuoles to strengthen tissue tolerance, thus avoiding damage to the cytoplasm, and the latter is often a more effective strategy in nonhalophytes. A high activity of the Na^+^ transport across the tonoplast in exchange for H^+^ is essential to reduce the Na^+^ toxicity in potato [8]. In this study, the Na^+^, K^+^, and Ca^2+^ flux treatment indicated that the efflux of Na^+^ in ‘Longshu 5’ was larger than that of ‘Qingshu 9’. At the same time, the stability of K^+^ can be maintained in ‘Longshu 5’, while the efflux of K^+^ was larger in ‘Qingshu 9’. A previous report showed that maintaining a high K^+^/Na^+^ ratio contributes to reducing the damage of salt to plants [48]. The salt-tolerant rice maintains a higher K^+^ level and coupled with the better antioxidant defense under salt treatments [12]. In addition, Ca^2+^ is essential in conferring salt tolerance and mediating salt-stress signal transduction [47], and an amount of Na^+^ receptor and transport protein perform function with the help of Ca^2+^. In this study, there was a stronger influx of Ca^2+^ in ‘Longshu 5’ than ‘Qingshu 9’, which showed there was abundant Ca^2+^ in the salt-tolerant potato. The difference of the ion flux may be one of the reasons for the stronger salt tolerance of ‘Longshu 5’ than ‘Qingshu 9’. These results showed that there were stronger capacities for the Na^+^ efflux, as well as K^+^ and Ca^2+^ ion uptake in the salt-tolerant potato cultivars, which provided key information for understanding the salt-tolerant mechanism in potato. Meanwhile, although ‘Qingshu 9’ is a drought-tolerant cultivar [31], our study showed that its ability to deal with salt ions was weak, indicating that the drought-tolerant cultivar may not tolerate salinity, and drought tolerance and salt tolerance may have different metabolic pathways, even considering that drought and salinity have common tolerance mechanisms. The results provide important information for us to further understand the mechanisms of salt tolerance and drought tolerance.

The SOS ion transporter pathway plays a vital role in the Na^+^ efflux and localization of vacuoles. Under salt stress, SOS genes are activated, and then downstream-related proteins are activated. The SOS pathway mainly involves five core components: the SCaBP8 (SOS3-LIKE CALCIUM BINDING PROTEIN8)–SOS2 complex, PKS5/14-3-3 complex, SOS1 (Sodium /hydrogen antiporter), AHX (Sodium /hydrogen exchanger), and ATPase [4]. The phosphorylation of SOS2 by PKS5 under normal conditions promotes the binding of SOS2 with 14-3-3 and inhibits the activity of SOS2 [49]. Meanwhile, PKS5/24 inhibits ATPase activity on the membrane [41]. Annexin (ANN) mediated early transients of Ca^2+^ signaling in the SOS pathway under salt stress with increasing Ca^2+^ levels in a short time [50]. When Ca^2+^ reaches a certain concentration, it binds with 14-3-3 to dissociate 14-3-3 from SOS2 and binds with PKS5. J3 and 14-3-3 jointly inhibit PKS5 kinase activity and eliminate the inhibition of SOS2 and membrane ATPase activity [41]. Under the action of the Ca^2+^ signal, SCaBP8 binds with SOS2 to form the SCaBP8–SOS2 complex, which inhibits the formation of ANN and maintains the intracellular Ca^2+^ concentration at a certain level [40]. A SCaBP8–SOS2 complex activates downstream target proteins SOS1, ATPase, and AHX. The plasma membrane ATPase and SOS1 cooperate to promote Na^+^ excretion to the outside of the cell, while vacuolar ATPase and AHX cooperate to promote Na^+^ excretion to the inside of the vacuole [41]. VIP1 transcription factor and CYP707A play a vital regulatory role in the salt-induced osmotic adjustment pathway [51,52]. In our study, the salt-tolerance cultivar was stronger in the capacities of osmotic adjustment and Na^+^ efflux, K^+^, and Ca^2+^ ion uptake. The expression levels of *StSOS1*, *StNHX4*, *StAKT1*, *StNAC24*, and *StCYP707A* were high under the NaCl treatment within 24 h and at different NaCl concentrations. Therefore, these studies revealed the important role of the ion transporter gene and NAC transcription factor, for example *StSOS1*, *StNHX4*, *StAKT1*, and *StNAC24*, to respond to salt stress in the salt-tolerance potato.

**Figure 7:**
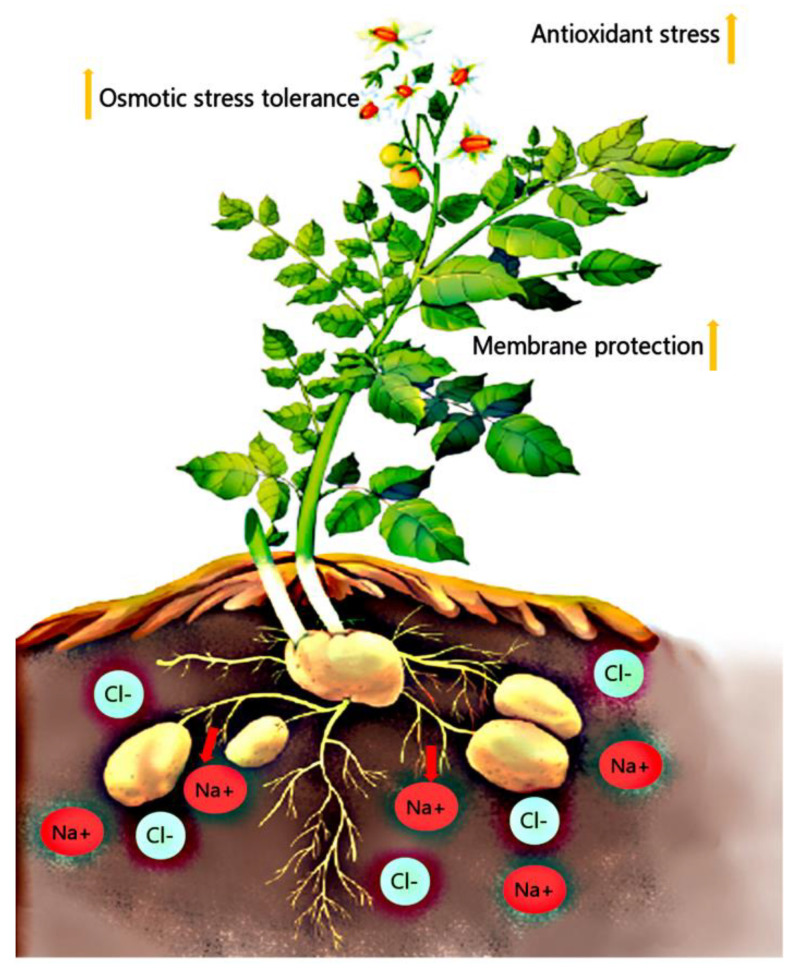
A hypothesis for the potato salt tolerance. The red arrow represents the Na^+^ efflux; the yellow arrows indicate enhancement effects.

We propose a hypothesis that the salt-tolerant potatoes resist salt stress by enhancing osmotic stress tolerance, increasing antioxidant capacity, and enhancing plasma membrane protection. Salt stress activates ion transporter gene expression, promotes Na^+^ ion efflux, and maintains the stability of K^+^ in the salt-tolerant potato. Meanwhile, the Ca^2+^ signal is active in the salt-tolerant potato, which may be beneficial to salt-stress signal transduction (Figure 7).

## 4. Materials and Methods

### 4.1. Plant Materials and Growth Conditions

The potato cultivars used in this study, ‘Longshu 5’ (salt-tolerance material) and ‘Qingshu 9’ (salt-susceptible material), were tetraploid cultivars with different salt tolerance screened from 52 materials that were exposed salt stress for 4 weeks (Figure S1). Tissue culture plantlets with the same growth for 4 weeks were chosen for this experiment. The culture conditions of the artificial tissue culture chamber were similar over the whole study at 22.5 ± 2 °C and 16 h of illumination (3000–5000 lx) per day.

### 4.2. Salt-Stress Treatments

#### 4.2.1. Selection of Appropriate Salt-Stress Concentration for Physiological Measurement

**Figure 8:**
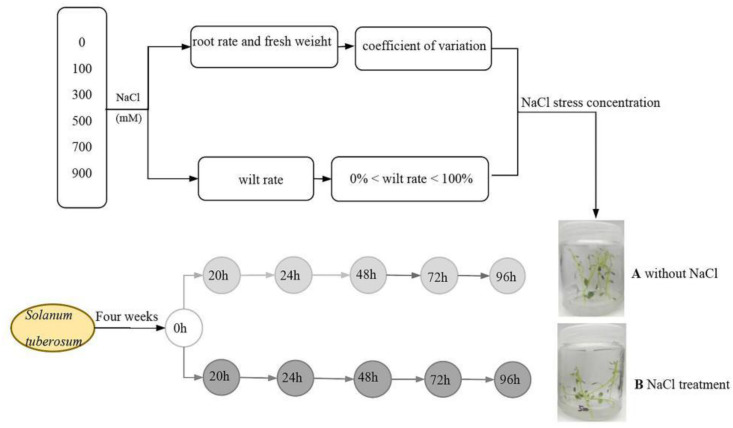
Schematic diagram of experimental design. A indicates MS medium without NaCl. B indicates MS medium containing the suitable stress concentration of NaCl.

The plants of the two cultivars containing 7 ± 1 stem segments were cultivated in Murashige and Skoog (MS) medium containing 0 mmol/L, 100 mmol/L, 300 mmol/L, 500 mmol/L, 700 mmol/L, and 900 mmol/L NaCl, with five plants in each replicate, and three biological replications. In order to make the phenotypes of the two cultivars differ in the short term, and to shorten the whole time of salt-tolerance identification, the salt concentration was reached in one time. The growth parameters of plants were measured after 96 h with light and dark during day, including the rooting rate, the fresh weight, and the wilt rate. The total coefficients of variation (*CV*) of the rooting rate and the fresh weight were calculated, and then the appropriate salt concentration was determined using the total coefficient of variation and 0% < wilt rate < 100% (Figure 8).

#### 4.2.2. Salt Treatment for Physiological Indicator Determination

A three-factor test design was performed. One factor was the cultivar: ‘Longshu 5’ and ‘Qingshu 9’; another factor was the salt concentration, including two levels, 0 and 500 mM NaCl; and the final factor was time of stress, including six levels, 0 h, 20 h, 24 h, 48 h, 72 h, and 96 h with light and dark during day. Seedlings were grown for four weeks and those at a similar stage from ‘Longshu 5’ and ‘Qingshu 9’ were chosen for this experiment. Stem segments (7 ± 1) from these seedlings were cultured without or with NaCl (appropriate stress concentration). Each replicate grew five stems with three replicates in total. After 0 h, 20 h, 24 h, 48 h, 72 h, and 96 h stress treatment, the contents of PRO, soluble protein, soluble sugar, and MDA, as well as activities of SOD, POD, and CAT, were measured.

#### 4.2.3. Salt Treatment for Na^+^, K^+^, and Ca^2+^ Flux Determination

The tissue culture plantlets of two cultivars with the same growth for 4 weeks were taken out from the solid MS medium and washed with deionized water. After cultivating with the liquid MS medium containing 0 mM and 200 mM NaCl in the test tube for 6 h [34], the Na^+^, K^+^, and Ca^2+^ flux in potato root meristem zone were measured. Each replication contains three plants, and a total of six replications.

#### 4.2.4. Expression Level of Ion Transport-Related Genes and Transcription Factors in ‘Longshu 5’ under NaCl

To explore the expression model of ion transport-related genes and transcription factors under different concentrations of NaCl stress, the tissue culture plantlets of stem segments (7 ± 1) from ‘Longshu 5’ were cultured in MS medium containing 0 mM and 200 mM NaCl with three biological replicates. After 0 h, 3 h, 6 h, 12 h, and 24 h, the total RNA of 27 treatments was extracted, respectively. In addition, the stem segments (7 ± 1) were cultured in MS medium containing 0 mmol/L, 50 mmol/L, 100 mmol/L, 150 mmol/L, and 200 mmol/L NaCl. Three biological replicates were set up with MS medium as control. After 24 h, with light and dark during day, total RNA of 27 treatments was extracted. Finally, all total RNA is reversely transcribed into cDNA.

### 4.3. Determination of Physiological and Biochemical Indicators

#### 4.3.1. Homogenized Protein Extraction

Fresh tissue (0.1 g) was weighed and homogenized in a centrifuge tube (1.5 mL) containing phosphate buffer solution (PBS, 50 mM, pH 7.8; fresh weight: buffer volume = 0.1 g: 1 mL) on ice [53]. For sufficient grinding, a small amount of quartz sand (about 15 grains) was added. The homogenate was centrifuged at 8000× *g* for 10 min at 4 °C, and the supernatant (crude protein extract) was removed and placed in a separate centrifuge tube for soluble protein and enzyme activity assays.

#### 4.3.2. Proline Content, Soluble Protein, and Soluble Sugar Determination

PRO was extracted with sulfosalicylic acid (SA) [54], according to the instructions of the Comin kit (Suzhou, China). Soluble protein was determined according to the instructions of the Comin kit (Suzhou, China). Finally, the absorptivity of the mixed liquid was determined at 562 nm [55] in a spectrophotometer. Soluble sugar was determined according to the instructions of the Comin Soluble Sugar Assay Kit (Suzhou, China). Refer to the method of Wang et al. [56].

#### 4.3.3. Determination of Antioxidant Enzyme Activity

The activities of CAT, POD, and SOD were assayed according to instructions of the respective kits (Comin, Suzhou, China). CAT decomposes H_2_O_2_, and H_2_O_2_ has a characteristic absorption peak at 240 nm [56]. Thus, the absorbance of the reaction solution at 240 nm decreases with the reaction time, and the CAT activity can be calculated according to the rate of change of absorbance. POD activity was determined by reference to the guaiacol method [56]. SOD activity was detected using xanthine and xanthine oxidase (XO) to generate O^2−^. O^2−^ reacted with nitro-blue tetrazolium (NBT) to form blue formazan, which has an absorption peak at 560 nm. SOD activity was determined by its 50% inhibition of the NBT reduction caused by the superoxides generated from the reaction of photoreduced riboflavin and oxygen. The results were expressed in units per milligram of plant fresh weight [57].

#### 4.3.4. MDA Content Determination

To assess the oxidative damage levels, the MDA content of plants was detected according to the manufacturer’s protocol (Comin, Suzhou, China). MDA content was measured by the thiobarbituric acid (TBA) reaction [57].

### 4.4. Principal Component Analysis

The average value of each index under the treatment of 0 and 500 mM NaCl were calculated. Then, the original data was transformed according to the salt tolerance coefficient formula:

Salt-tolerance coefficient [17] = Values under stress/Values under control × 100%

Secondly, the above salt-tolerance coefficient was standardized, and then the PCA was applied [21]. PCs were extracted according to the principle that the eigenvalues were above 1 and the cumulative contribution rate was more than 80%. The extracted PCs can represent the initial standardized indicator via the following principal component formula:

PCA1 = A_11_X_1_ + A_12_X_2_ + A_13_X_3_ + …… + A_1P-1_X_P-1_ + A_1P_X_P_

PCA2 = A_21_X_1_ + A_22_X_2_ + A_23_X_3_ + …… + A_2P-1_X_P-1_ + A_2P_X_P_

PCAm = A_m1_X_1_ + A_m2_X_2_ + A_m3_X_3_ + …… + A_mP-1_X_P-1_ + A_mP_X_P_

where Ai1, Ai2, Ai3, ……, Amp (I = 1, ……, m) are the eigenvectors corresponding to the eigenvalue of the covariance matrix of X, and X1, X2, ……, X_P_ are the standardized values of the original indicators (the standardization of raw data is in order to eliminate the impact of dimension). Finally, the comprehensive evaluation can be implemented through composite scores from the evaluation function as below: Composite score = summer (Fi × Wi) (Fi is principal component score; Wi is percentage of variance of the PCAi)

### 4.5. Ca^2+^, Na^+^, and K^+^ Flux Determination

Ca^2+^, Na^+^, and K^+^ flux assays were conducted with Non-invasive Micro-test Technology (NMT). Net fluxes were obtained with the Physiolyzer^®^ NMT system (Younger USA) at the Xuyue Beijing NMT Research Service Center, China. Potato, including ‘Longshu 5’ and ‘Qingshu 9’ grown for four weeks in MS medium, were stressed with 200 mM NaCl or grown without NaCl for 6 h, and then the Na^+^, K^+^, and Ca^2+^ flux of the meristem zone (200 mm from the root tip) of the root were recorded with 10 min of equilibration in 5 mL testing solution (testing solution components: 0.1 mM KCl, 0.1 mM CaCl_2_, 0.1 mM MgCl_2_, 0.5 mM NaCl, 0.3 mM MES, 0.2 mM Na_2_SO_4_, pH 6.0) by Xuyue (Beijing) Sci. and Tech. Co., Ltd., Beijing, China using the NMT. Six roots in each repetition were tested with 5 min stability data. Ca^2+^, Na^+^, and K^+^ flux was measured using imFluxesV2.0 (Xuyue (Beijing) Sci. and Tech. Co., Ltd., Beijing, China).

### 4.6. Quantitative Real-Time PCR (qRT-PCR) of Ion Transport-Related Genes

To explore the expression level of ion transport-related genes and transcription factors under NaCl stress, qRT-PCR was performed for 7 genes. The qRT-PCR protocol used here is described in a previous study [19]. All primers used in this study were designed using Primer 5.0.

### 4.7. Statistical Analysis

Data were statistically analyzed in SPSS v. 22.0 (IBM SPSS Statistics Inc., Chicago, IL, USA) and presented as means ± standard deviation (SD). Duncan multiple comparisons was used to analyze the significance of physiological indicators under different NaCl stress and stress duration. One-way analysis of variance was used determine whether there were significant differences in gene expression levels under different stress times or NaCl stress concentrations. For all statistical analyses, 0.05 was set as the significance level. The principal components were extracted, and scores were calculated using SPSS v. 22.0.

## 5. Conclusions

The proline content, soluble protein, soluble sugar, and MDA, and the activities of CAT, POD, and SOD, changed significantly under different salt concentrations and time lengths of stress. Therefore, these seven physiological indicators can be used to rapidly identify the salt tolerance of potato. Moreover, soluble protein and soluble sugar responded more rapidly than proline to salt stress. The principal component analysis verified that the salt tolerance of ‘Longshu 5’ was stronger than that of ‘Qingshu 9’. The strong efflux of Na^+^, the stability of K^+^, and the absorption of Ca^2+^ may be the key reasons for stronger salt tolerance of ‘Longshu 5’, which indicated the ability to mitigate ion toxicity and play a more crucial role in tolerating salinity in potato. Ion transport genes *StSOS1*, *StNHX4*, *StAKT1*, and *StCYP707A* may contribute to the salt tolerance of potato. Especially, the *StCYP707A* gene can respond to long-term high-salinity stress.

## Figures and Tables

**Table 1:** Growth parameter analysis under different salt shock concentrations within 96 h.

Salt Concentration (mmol/L)	Root Percentage (%)		Fresh Weight (g)		Total CV(%)	Wilt Rate (%)	
	Longshu 5	Qingshu 9	Longshu 5	Qingshu 9		Longshu 5	Qingshu 9
0	100	100	0.715	2.678	57.6	0.0	0.0
100	100	100	0.555	1.483	32.5	0.0	0.0
300	0.0	0.0	0.442	1.306	122.0	0.0	0.0
500	0.0	0.0	0.414	1.227	122.1	26.67	33.33
700	0.0	0.0	0.385	0.917	115.5	100	100
900	0.0	0.0	0.333	0.746	113.7	100	100

Note: CV, variable coefficient.

**Table 2:** Effect of NaCl stress on MDA content.

Variety	NaCl Concentration (mmol/L)	MDA Content (nmol/g)					
		0 h	20 h	24 h	48 h	72 h	96 h
Longshu 5	0	8.69 ± 0.91 ^d^	9.80 ± 1.36 ^bcd^	8.60 ± 0.59 ^d^	9.89 ± 0.29 ^bcd^	9.29 ± 0.1 ^cd^	9.12 ± 0.40 ^cd^
	500	8.69 ± 0.91 ^d^	9.80 ± 0.93 ^bcd^	9.89 ± 1.05 ^bcd^	10.75 ± 0.39 ^bc^	11.10 ± 0.93 ^b^	12.90 ±1.29 ^a^
Qingshu 9	0	12.51 ±1.16 ^b^	11.86 ± 0.78 ^b^	12.39 ± 1.34 ^b^	12.25 ± 1.42 ^b^	12.90 ± 1.29 ^b^	11.22 ± 0.13 ^b^
	500	12.51± 1.16 ^b^	10.92 ± 1.27 ^b^	13.07 ± 1.30 ^b^	16.17 ± 0.91 ^a^	12.38 ± 0.78 ^b^	11.61 ± 1.80 ^b^

Note: MDA, malondialdehyde. Data are expressed as the means±SD. Values with different letters in the same row are significantly different at *p* < 0.05 according to Duncan test (*n* = 3). Same lowercase letters mean no significant difference, different lowercase letters mean significant difference.

**Table 3:** Eigenvalue and contribution rate of seven test indicators.

Component	Initial Eigenvalues			Extraction Sums of Squared Loadings		
	Total	% of Variance	Cumulative %	Total	% of Variance	Cumulative %
PCA1	3.144	44.912	44.912	3.144	44.912	44.912
PCA2	1.716	24.511	69.423	1.716	24.511	69.423
PCA3	1.025	14.639	84.062	1.025	14.639	84.062
PCA4	0.636	9.089	93.151			
PCA5	0.285	4.078	97.229			
PCA6	0.136	1.937	99.166			
PCA7	0.058	0.834	100			

Note: PCA refers to the principal component.

**Table 4:** Principal component and composite scores of the two cultivars.

Cultivar	Time (h)	Principal Component Scores			Composite Scores	Total Score
		PCA 1	PCA 2	PCA 3		
**Longshu 5**	0	−0.245	−0.280	−1.376	−0.337	
	20	1.139	−0.197	−0.362	−0.002	
	24	0.758	−0.886	1.439	0.374	
	48	−0.220	−0.818	0.679	0.058	1.703
	72	1.246	−0.173	0.115	0.805	
	96	1.211	1.072	−1.177	0.805	
**Qingshu 9**	0	−0.245	−0.280	−1.376	−0.337	
	20	−0.498	−0.698	−0.433	−0.386	
	24	0.133	−0.841	1.025	0.467	−1.703
	48	−2.130	−0.531	0.059	0.015	
	72	−1.063	1.591	0.024	−0.649	
	96	−0.087	2.040	1.383	−0.813	

Note: PCA, principal component.

## Data Availability

The data presented in this study are available on request from the corresponding author. The data are not publicly available due to privacy.

## Supplementary Materials

The following supporting information can be downloaded at: https://www.mdpi.com/article/10.3390/plants11121565/s1, Figure S1: Potato cultivars salt stress exposure for 4 weeks; Table S1: Salinity-tolerance coefficient of different indicators; Table S2: Correlation matrix of seven indicators; Table S3: Characteristic vectors of normalization by principal component analysis.
