# Supplementary material for: Physiology and Gene Expression Analysis of Potato (Solanum tuberosum L.) in Salt Stress
Source: Plants (Basel). 2022 Jun 14;11(12):1565. doi: 10.3390/plants11121565 (PMC9229698; doi:10.3390/plants11121565)
Supplement: Supplementary file 1 [file plants-11-01565-s001.zip › Supplementary tables and figure.pdf]

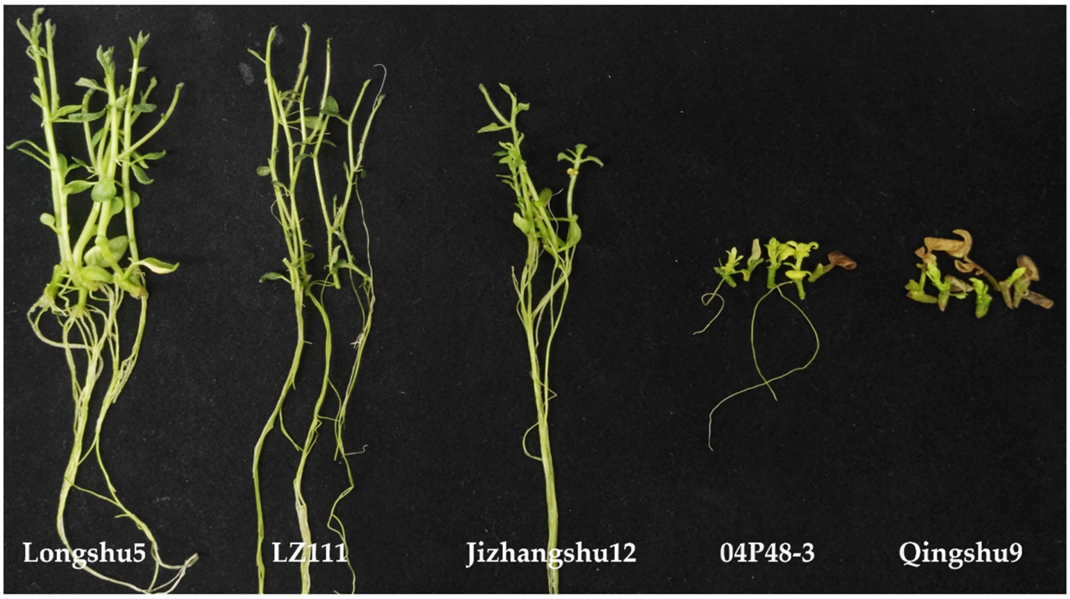

Figure S1. Potato cultivars salt stress exposure for 4 weeks.

Table S1. Salinity tolerance coefficient of different indicators.

Cultivar	Time (h)	Salinity tolerant coefficient						
		Proline content	Soluble protein	Soluble sugar	CAT activity	POD activity	SOD activity	MDA content
'Longshu 5'	0	1	1	1	1	1	1	1
	20	1.201	1.411	1.170	0.890	0.919	1.434	1
	24	0.888	1.453	1.967	0.713	1.014	1.507	1.150
	48	4.804	1.322	1.192	0.822	1.288	1.201	1.087
	72	9.464	1.501	1.825	0.779	1.661	1.807	1.194
	96	13.101	1.295	1.854	0.891	1.441	1.672	1.415
CV (%)		92.7	12.3	25.8	10.8	21.9	18.9	12.4
'Qingshu 9'	0	1	1	1	1	1	1	1
	20	1.429	1.102	1.058	0.915	1.084	0.979	0.920
	24	3.846	1.818	2.008	0.713	1.232	1.360	1.055
	48	6.497	1.061	1.664	0.875	1.196	0.624	1.319
	72	20.424	0.959	1.751	0.763	1.164	0.768	0.960
	96	26.545	0.751	1.049	0.706	1.212	1.046	1.035
CV (%)		99.3	29.9	28.1	13.2	7.1	23.9	12.3

Note：*CV* refers to the coefficients of variation.

Table S2. Correlation matrix of seven indicators.

	Zproline content	Zsoluble protein	Zsoluble sugar	ZCAT activity	ZPOD activity	ZSOD activity	ZMDA content
**Zproline content**	1						
**Zsoluble protein**	-0.463	1					
**Zsoluble sugar**	0.111	0.615*	1				
**ZCAT activity**	-0.497*	-0.327	-0.588*	1			
**ZPOD activity**	0.407	0.271	0.481	-0.343	1		
**ZSOD activity**	-0.126	0.686**	0.411	-0.255	0.476	1	

**ZMDA content**	0.113	0.229	0.568	-0.077	0.575*	0.361	1

Note: Z refers to the standardized data via Z score. * and ** indicated a significant difference significant difference ($p<0.05$) and extremely significant difference ($p<0.01$), respectively.

Table S3. Characteristic vectors of normalization by principal component analysis.

	**Component**		
	**PCA 1**	**PCA 2**	**PCA 3**
**Zproline**	0.095	-0.728	-0.049
**Zsoluble protein**	0.395	0.479	-0.263
**Zsoluble sugar**	0.483	-0.006	-0.131
**ZCAT**	-0.345	0.315	0.629
**ZPOD**	0.421	-0.245	0.337
**ZSOD**	0.41	0.281	0.03
**ZMDA**	0.369	-0.055	0.633
